# Adropin inhibits the progression of atherosclerosis in ApoE^-/-^/Enho^-/-^ mice by regulating endothelial-to-mesenchymal transition

**DOI:** 10.1038/s41420-023-01697-3

**Published:** 2023-10-31

**Authors:** Teng Ying, LingZhen Wu, TingXiang Lan, ZhiXiong Wei, DanQing Hu, YiLang Ke, Qiong Jiang, Jun Fang

**Affiliations:** 1 Department of Cardiology, Fujian Medical University Union Hospital; Fujian Cardiovascular Medical Center; Fujian Institute of Coronary Artery Disease; Fujian Cardiovascular Research Center, Fuzhou, PR China; 2 Department of Cardiology, The First Affiliated Hospital of Jiangxi Medical College, Shangrao, PR China; 3 Department of Ultrasound, Longyan First Hospital Affiliated to Fujian Medical University, Longyan, PR China; 4 School of Health, Fujian Medical University, Fuzhou, PR China; 5 Department of Geriatrics, Fujian Medical University Union Hospital; Fujian Key Laboratory of Vascular Aging, Fujian Institute of Geriatrics, Fuzhou, PR China

**Keywords:** Cell biology, Pathogenesis

## Abstract

Adropin, a secreted protein, coded by energy homeostasis-associated gene (Enho), is recently reported to modulate atherogenesis, with endothelial-to-mesenchymal transition (EndMT) involved in the early process. We explored whether adropin may alleviate atherosclerosis by regulating EndMT. We found that an intraperitoneal injection of adropin [105 μg/(kg·d) for 13 weeks] inhibited the progression of high-fat diet (HFD)-induced aortic atherosclerosis in apolipoprotein E-deficient mice (ApoE^–/–^) and those with double gene deletion (ApoE^–/–^/Enho^–/–^), as detected by Oil Red O and haematoxylin-eosin staining. In the aortas of ApoE^–/–^ mouse, adropin treatment ameliorated the decrease in the mRNA expression of endothelial cell markers (leukocyte differentiation antigen 31, CD31, and vascular endothelial cadherin, VE-cadherin), but increased that of EndMT markers (alpha smooth muscle actin, α-SMA, and fibroblasts specific protein-1). In vitro, an adropin treatment (30 ng/ml) arrested the hydrogen peroxide (H_2_O_2_)-induced EndMT in human umbilical vein endothelial cells (HUVECs), attenuated the morphological changes of HUVECs, reduced the number of immunofluorescence-positive α-SMA, increased the mRNA and protein expressions of CD31 and VE-cadherin, and decreased those of α-SMA. Furthermore, the adropin treatment decreased the mRNA and protein expressions of transforming growth factor (TGF)-β1 and TGF-β2, and suppressed the phosphorylation of downstream signal protein Smad2/3 in HUVECs. These mitigative effects of adropin on H_2_O_2_-induced EndMT were reversed by the transfection of TGF-β plasmid. The findings signify that adropin treatment may alleviate the atherosclerosis in ApoE^–/–^/Enho^–/–^ mice by inhibiting EndMT via the TGF-β/Smad2/3 signaling pathway.

## Introduction

Endothelial dysfunction is involved in the initial stage of atherosclerosis, in which the endothelial-to-mesenchymal transition (EndMT) plays a significant role in the early development of atherosclerotic lesions [1–3]. The occurrence of EndMT may increase the vascular endothelial permeability and accumulation of lipid in the subintima, which promotes the development of chronic inflammation and atheromatosis [1–3].

The process of EndMT can also change the morphology of endothelial cells, resulting in the loss of their barrier function. The aggravation of EndMT can dramatically decrease the expressions of leukocyte differentiation antigen (CD) 31 and vascular endothelial cadherin (VE-cadherin), which are the markers of morphology and function of endothelial cells. Meanwhile, the occurrence of EndMT will induce a gradual expression of alpha smooth muscle actin (α-SMA), smooth muscle 22 alpha (SM22α), and fibroblasts specific protein (FSP)-1, which are usually not expressed in endothelial cells. Therefore, the expressions of these markers can be manipulated to detect whether the endothelial cells have been converted into mesenchymal cells [4, 5].

Available literature suggests that reducing EndMT may be an important strategy to mitigate the occurrence and development of atherosclerosis [1–3]. Studies have documented that endothelial cells can express endogenous protective factor called adropin, a newly discovered secretory protein coded by energy homeostasis-associated gene (*Enho*) that plays an important role in maintaining energy balance, fat metabolism, and insulin response [6, 7]. In vitro experiments have reported that adropin can regulate the function of endothelial cells by increasing endothelial nitric oxide synthase [8]. Our previous studies evidence that adropin may offer cardioprotection against the myocardial ischemia-reperfusion injury in rodents [9] and that a low level of serum adropin may increase the risk of coronary heart disease and the severity of coronary atherosclerosis [10]. A recent study reports that adropin can contribute to anti-atherosclerosis in apolipoprotein E-deficient mouse (ApoE^-/-^) mice with high fat diet (HFD) by suppressing monocyte-endothelial cell adhesion and smooth muscle cell (SMC) proliferation [11]. However, it remains unelucidated with regards to the potential mechanisms underlying the involvement of EndMT in adropin-induced anti-atherosclerosis.

Given the role of EndMT in atherosclerosis and the protective effect of adropin on endothelial cells, we attempted to determine the role of EndMT in adropin-induced anti-atherosclerosis using the HFD-fed ApoE^-/-^ and ApoE^-/-^/Enho^-/-^ double-gene-knockout (DKO) mouse models and the in vitro hydrogen peroxide (H_2_O_2_)-induced EndMT model. We found that adropin treatment downregulated the expression of TGF-β and phosphorylation of Smad2/3, inhibited EndMT, and alleviated arteriosclerosis in ApoE^-/-^/Enho^-/-^ mice. These findings may provide novel insights into the potential mechanism underlying the involvement of EndMT in adropin-induced anti-atherosclerosis.

## Results

### Adropin in vivo suppresses atherosclerosis in ApoE^-/-^ mice and ApoE^-/-^/Enho^-/-^ mice

**Fig. 1:**
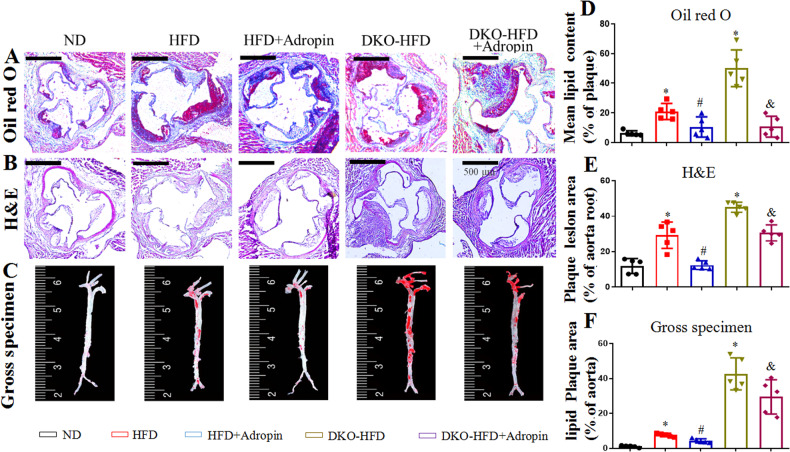
The alleviated atherosclerosis in ApoE^-/-^ mice and ApoE^-/-^/Enho^-/-^ mice by adropin treatment. **A** Cross-sections of the aortic roots were stained by ORO, red part showing lipid in plaque. **B** H&E staining from the same samples by ORO staining, revealing plaque tissues protruding from lumen. **C** Gross morphology of the whole aortas, the red parts indicating lipid plaque by ORO staining. **D** The result of ORO staining indicated as a percentage of lipid area/plaque area. **E** The result of H&E staining was shown as a percentage of plaque area/lumen area. **F** The result of ORO staining in the whole aortas was shown as a percentage of lipid plaque area/aorta area. The above results revealed that the aggravated atherosclerosis in ApoE^-/-^ mice or ApoE^-/-^/Enho^-/-^ mice fed with high fat diet was reversed by the adropin treatment. ND, the normal diet group. HFD, the high fat diet group. HFD+Adropin, the group fed with HFD together with Adropin treatment. DKO-HFD group: ApoE^-/-^/Enho^-/-^ double-gene-knockout (DKO) mice were fed with HFD. DKO-HFD+Adropin group: DKO mice were fed with HFD together with Adropin. H&E, haematoxylin-eosin. The data are presented as mean ± SEM. and analyzed by ANOVA. *n* = 5. ^*^P < 0.05, as compared with ND; ^#^ *P* < 0.05, as compared with HFD; ^&^ *P* < 0.05, as compared with DKO-HFD. Bar = 500 μm in (**A**) and (**B**), and 10 mm in **C**.

ORO and H&E staining were performed to evaluate the severity of aortic atherosclerosis in ApoE^-/-^ mice fed with HFD. The aortic roots of mice were stained with ORO and H&E. ORO staining showed that the HFD group reported more lipid accumulation in arterial plaques than that the ND group [(21 ± 2)% vs. (6 ± 1)%, *P* < 0.05]. However, the adropin treatment (the HFD + Adropin group) significantly lowered the lipid accumulation [(10 ± 3)%, *P* < 0.05] when compared with the HFD group. In addition, the double gene knockout mice (DKO-HFD group) showed the most lipid accumulation among all the groups, which was significantly decreased by adropin treatment [(50 ± 6)% vs. (10 ± 3)%, *P* < 0.05], indicating that adropin can reduce the accumulation of lipid under intima (Fig. 1A, D).

Similarly, H&E staining showed no obvious atherosclerotic plaques in the ND group but obvious atherosclerotic plaques in the DKO-HFD mice [(12 ± 2)% vs. (45 ± 1)%, *P* < 0.05]. The HFD group featured obvious atherosclerotic plaques that protruded on the surface of intima [(29 ± 3)%, *P* < 0.05]. Compared with the DKO-HFD+Adropin group, the HFD + Adropin group displayed only local bulging plaques on the intimal surface with complete fiber caps at the top of the plaques [(12 ± 1)% vs. (31 ± 2)%, *P* < 0.05] (Fig. 1B, E). These findings suggest that adropin may significantly alleviate the severity of atherosclerosis. Furthermore, the results of gross morphology in aortic arches were consistent with those in aortic roots by ORO and H&E staining, further indicating the reduced atherosclerosis by adropin treatment (Fig. 1C, F).

Despite the importance of blood lipid in atherosclerosis, we found that adropin imposed no impact on the blood lipid and body weight in the ApoE^-/-^ mice (Supplementary Fig. 2).

### Adropin in vivo inhibits the aortic EndMT in ApoE^-/-^ mice

**Fig. 2:**
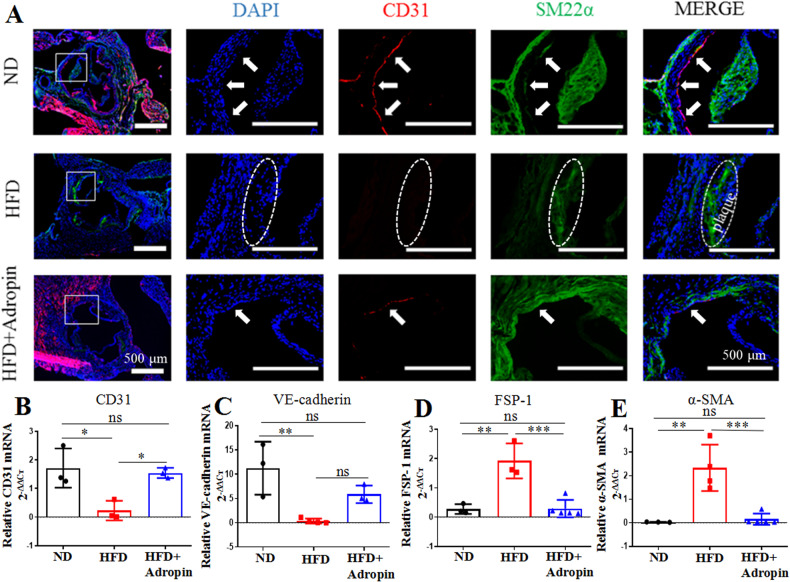
The qualitative and quantitative analyses of endothelial-to-mesenchymal transition (EndMT). **A** The leftmost column: a complete view of cross-sections of the aortic roots, with the rectangle indicating the sites where the amplified pictures in other four column came from. The left second column (DAPI, blue): the nuclei of 4’,6-diamidino-2-phenylindole positive cells. The median column (endothelial cell marker CD31, red): the complete expression of CD31 was showed in aortic intima (arrowhead) in the ND group, and HFD led to absence in the expression of CD31, which was rescued by adropin treatment (the HFD+Adropin group). The right second column (mesenchymal cell marker SM22α, green): oval box showed that HFD induced obvious plaque formation and mass expression of SM22α with no obvious CD31 expression in aortic intima, indicating the occurrence of EndMT, which was reversed by adropin treatment. **B**–**E** The statistical histograms of mRNA expressions from endothelial cell markers CD31 and VE-cadherin, and mesenchymal cell markers α-SMA and FSP-1. The rightmost column: merged images. CD31, leukocyte differentiation antigen 31. VE-cadherin, vascular endothelial cadherin. α-SMA, alpha smooth muscle actin. FSP, fibroblasts specific protein-1. SM22α, smooth muscle 22 alpha. DAPI, 4’,6-diamidino-2-phenylindole. Bar = 500 μm. The data are presented as mean ± SEM. and analyzed by ANOVA. *n* = 3–5. *P < 0.05, **P < 0.01, ***P < 0.001.

The immunofluorescence double staining of CD31 and SM22α in consecutive aortic frozen sections evidenced the presence of EndMT in the in vivo process of atherosclerosis under the confocal microscope (Fig. 2A). This qualitative result showed that the ND group reported a continuous expression of CD31 in vascular endothelial cells but no obvious expression of SM22α. The HFD produced a significant absence in the expression of CD31 and an increase in the expression of SM22α in the inner vascular wall, with obvious plaque formation in the HFD group. Compared with the HFD group, the adropin treatment yielded a higher expression of CD31 and a lower expression of SM22α in the HFD+Adropin group, though the ND group reported the highest expression of CD31 and lowest expression of SM22α.

Furtherly, q-PCR was performed to quantify the gene expressions of EndMT-related factors in the aortic inner wall, including the markers of endothelial cells (CD31 and VE-cadherin) and the markers of mesenchymal cells (α-SMA and FSP-1) (Fig. 2B–E). The HFD decreased the mRNA expressions of CD31 and VE-cadherin but increased the mRNA expressions of α-SMA and FSP-1, which were reversed by the adropin treatment. Taken together, these qualitative and quantitative results suggest that adropin may protect the endothelial cells against EndMT by maintaining their morphological and functional integrity.

### Adropin in vitro inhibits the conversion of HUVECs into smooth muscle cells

**Fig. 3:**
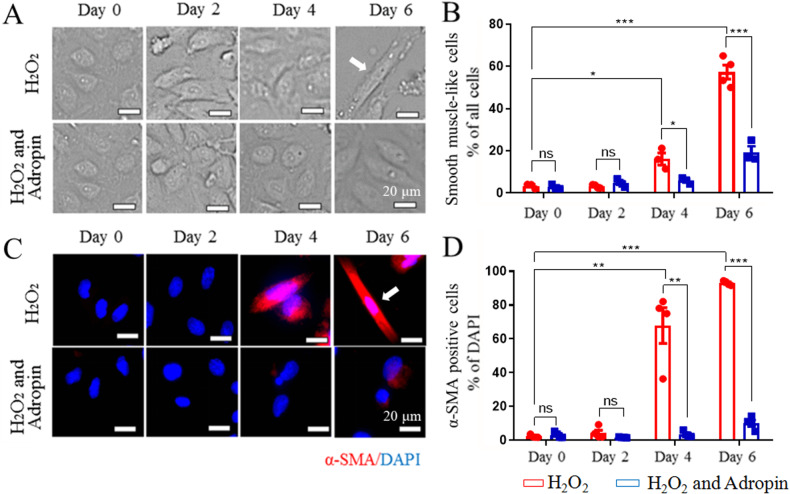
The inhibited hydrogen peroxide (H_2_O_2_)-induced conversion of HUVECs into smooth muscle cells (SMCs) by adropin treatment. **A** H_2_O_2_-induced morphological changes of HUVECs at different timepoints. Fusiform cells were considered as smooth muscle-like cells after 4 days of culture (arrowhead). **B** The percentage of fusiform smooth muscle-like cells in the total HUVECs. **C** H_2_O_2_-induced α-SMA positive expression in HUVECs at different timepoints by immunofluorescence. Fusiform α-SMA positive cells were marked red (arrowhead). **D** The percentage of α-SMA positive cells (red) /DAPI positive cells (blue). Adropin treatment (the H_2_O_2 _+ Adropin) alleviated H_2_O_2_-induced SMCs-like change (**A**, **B**) and expression of mesenchymal cell marker α-SMA (**C**, **D**) in HUVECs, suggesting the reversal of EndMT. HUVECs, human umbilical vein endothelial cells. α-SMA, alpha smooth muscle actin. DAPI, 4’,6-diamidino-2-phenylindole. EndMT, Endothelial-to-mesenchymal transition. The data are presented as mean ± SEM. and analyzed by *t* tests. *n* = 4. *P < 0.05, **P < 0.01, ***P < 0.001. Bar = 20 μm.

Intuitively, SMCs in atherosclerotic plaques may be converted from endothelial cells, an in vitro H_2_O_2_-induced EndMT model was further adopted to evaluate the effects of adropin on the conversion. Light microscopy revealed smooth muscle-like cells due to the H_2_O_2_-induced morphological changes of human umbilical vein endothelial cells (HUVECs). The results showed that after 4 days of culture, H_2_O_2_ significantly changed the morphology of endothelial cells into the fusiform shape, producing smooth muscle-like cells (Fig. 3A). Compared with the H_2_O_2_ group, adropin treatment protected the morphology of endothelial cells from the SMCs-like change, indicated as a decrease in the proportion of SMCs-like cells in the H_2_O_2 _+ Adropin [day 6, (57 ± 6)% vs. (3 ± 1)%, *P* < 0.001] (Fig. 3B).

Immunofluorescence was performed to detect the proportion of α-SMA-positive cells in HUVECs (Fig. 3C, D). As α-SMA, a marker of SMCs, is not expressed in normal HUVECs, we adopted the H_2_O_2_ treatment to induce the morphological change of HUVECs and the adropin administration to inhibit the morphological conversion, and detected the presence of the specific markers of SMCs. The severity of EndMT was assessed by calculating the positive rate of α-SMA. The analyses showed that the expression of α-SMA increased with the prolongation of H_2_O_2_ treatment, which was blocked by the adropin treatment [day 6, (93 ± 1)% vs. (10 ± 4)%, *P* < 0.001] (Fig. 3D). The results evidence that adropin treatment alleviates H_2_O_2_-induced EndMT.

### Adropin in vitro maintains the expressions of CD31 and VE-cadherin, and inhibits the expression of α-SMA in HUVECs

**Fig. 4:**
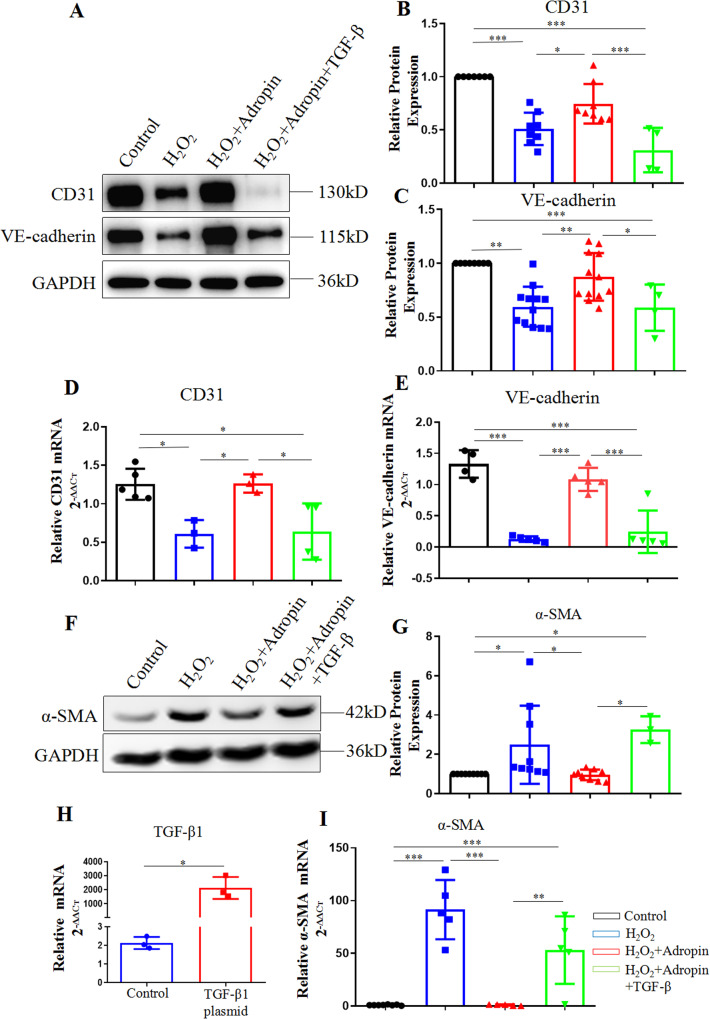
The maintained gene and protein expressions of CD31,VE-cadherin TGF-β1 and α-SMA in HUVECs by adropin treatment. **A**–**C** Western blotting results of CD31 and VE-cadherin. **D**, **E** q-PCR results of CD31 and VE-cadherin. **F**, **G** Western blotting results of α-SMA. **H** q-PCR results of TGF-β1. **I** q-PCR results of α-SMA. The data are presented as mean ± SEM. and analyzed by ANOVA. *n* = 3–7. *P < 0.05, **P < 0.01, *P < 0.001.

As specific proteins of endothelial cells, the decline in the expressions of CD31 and VE-cadherin strongly indicates a decrease in endothelial cells. Meanwhile, the occurrence of EndMT can induce an overexpression of mesenchymal cell markers. Given the morphological changes, western blotting and q-PCR were adopted to analyze the gene and protein expressions of CD31, VE-cadherin and α-SMA in HUVECs (Fig. 4A–G, I, Supplementary Fig. 3). The transfection TGF-β1 plasmid was also performed to explore the role of the TGF-β pathway. q-PCR reported a highly efficient transfection of TGF-β1 plasmid when compared with the Control group [(2134.9 + 786.4) vs. (2.1 + 0.3), *P* < 0.001] (Fig. 4H).

Compared with the Control group, H_2_O_2_ (the H_2_O_2_ group) led to a lower gene and protein expressions of CD31 and VE-cadherin, but a higher expression of α-SMA, indicating significant EndMT, which was improved by the adropin treatment (the H_2_O_2 _+ Adropin group). However, the adropin-induced increase in the gene and protein expressions of CD31 and VE-cadherin and that in protein expression of α-SMA were blocked by the transfection of TGF-β1 plasmid into HUVECs (the H_2_O_2_ + Adropin + TGF-β group). These results suggest that adropin can maintain the expressions of CD31 and VE-cadherin but inhibit the expression of α-SMA in HUVECs, and that the TGF-β signaling pathway may be involved in adropin-induced attenuation of EndMT.

### Adropin in vitro inhibits the TGF-β/Smad2/3 pathway in HUVECs

**Fig. 5:**
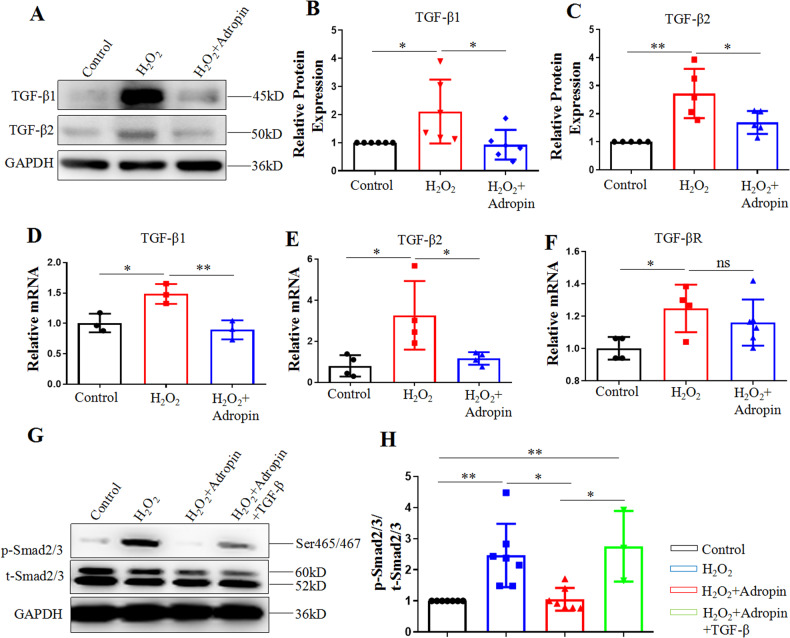
The inhibited TGF-β/Smad2/3 pathway in vitro in HUVECs by adropin treatment. **A**–**C** Western blotting results of TGF-β1 and TGF-β2. **D**, **E** q-PCR results of TGF-β1 and TGF-β2. **F** q-PCR results of TGF-βR. **G**, **H** Western blotting results of total and phosphorylated Smad2/3. HUVECs, human umbilical vein endothelial cells. TGF-β, transforming growth factor-β. TGF-βR, transforming growth factor-β receptor. The data are presented as mean ± SEM. and analyzed by ANOVA. *P < 0.05, **P < 0.01.

We further explored the possible molecular mechanism underlying the adropin-induced attenuation of EndMT. The gene and protein expressions of TGF-β1 and TGF-β2 were analyzed by western blotting and q-PCR. Compared with the Control group, H_2_O_2_ (the H_2_O_2_ group) increased the mRNA and protein expression of TGF- β2 and the mRNA expression of TGF-β1, which were significantly decreased by adropin treatment (the H_2_O_2_ + Adropin group) (*P* < 0.05) (Fig. 5A, C–E, Supplementary Fig. 3). Compared with the Control group, H_2_O_2_-induced protein expression of TGF-β1 only showed an upward trend without a statistical significance, which was downregulated by the adropin treatment (*P* < 0.05) (Fig. 5B). We further investigated whether the adropin treatment impacted the gene expression of TGF-β receptor (TGF-βR). q-PCR results showed that the adropin treatment failed to inhibit the H_2_O_2_-induced upregulation of mRNA expression of TGF-βR (Fig. 5F). These results indicate that adropin may exert anti-EndMT and anti-atherosclerosis effects by directly inhibiting the expressions of TGF-β1 and TGF-β2, and indirectly acting on TGF-βR and its related downstream factors.

As a downstream signal protein of TGF-β, Smad2/3 protein is closely related to the occurrence of EndMT. Therefore, we analyzed the total and phosphorylated protein expression of Smad2/3 (p-Smad2/3/t-Smad2/3) by Western blotting (Fig. 5G, H). Compared with the Control group, H_2_O_2_ increased the phosphorylation of Smad2/3, which was downregulated by the adropin treatment (*P* < 0.05). The adropin-induced downregulation of p-Smad2/3 was blocked by the transfection of TGF-β1 plasmid (the H_2_O_2_ + Adropin + TGF-β group). These data suggest that the TGF-β/Smad2/3 signaling pathway may be involved in the adropin-induced attenuation of EndMT.

## Discussion

In this study, we provided more reliable evidence for the anti-atherosclerotic effects of adropin by using ApoE^-/-^/Enho^-/-^ mice, and further demonstrated that adropin inhibited the occurrence and development of atherosclerosis in high-fat diet (HFD)-fed ApoE^-/-^ mice by inhibiting the EndMT. Moreover, the anti-atherosclerotic effect of adropin was achieved by inhibiting the expression of TGF-β and weakening the phosphorylation of Smad2/3 in endothelial cells. These findings provide some novel insights into the therapeutic value of adropin for the treatment fo atherosclerosis.

Adropin, composed of 76 amino acid residues, is a new secretory protein discovered by Kumar et al. in 2008 [6]. It is encoded by intracellular *Enho* gene and is expressed and secreted in the liver, brain, coronary arterial endothelium and other tissues and organs [6, 8]. Availble studies evidence that adropin is closely related to pro-atherosclerotic metabolic disorders, such as, lipid metabolism disorder, obesity, and metabolic syndrome [6, 7, 10].

Recent studies have reported that adropin, at a dose of 10 μg/kg/h (240 μg/kg/d) using subcutaneously osmotic minipumps for 4 weeks, may confer anti-atherosclerotic effects in ApoE^-/-^ mice receiving HFD [11]. In this study, we established atherosclerotic models of ApoE^-/-^ mice receiving HFD and intraperitoneal injection of adropin from 8 weeks of age for 13 weeks, with the 13-week HFD scheme chosen according to a previous report [12]. The intraperitoneal doses of adropin used in the studies for acute metabolic regulation varied from 2.1 μg/kg/d (once a day, for 10 days) to 2000 μg/kg (3–5 injections) [13–15]. However, the effective dose of adropin for chronic administration has not been well established. In this study, a median dose of adropin (105 μg/kg/d) was used for a long-term intraperitoneal injection (for 13 weeks from 8 weeks of age). Nevertheless, despite the differences in the dose of adropin, the age of animals and the duration of HFD, we found that adropin at a dose of 105 μg/kg/d for 13 weeks significantly reduced the area of plaques and accumulation of lipid in aortas. Furthermore, the current study employed a double gene knockout animal model (the ApoE^-/-^/Enho^-/-^ mice) that has a severer atherosclerosis and found that the supplementation of adropin significantly ameliortated the above effects. Altogether, these findings strongly suggest that adropin may exert anti-atherosclerotic effects in the ApoE^-/-^ mice and the ApoE^-/-^/Enho^-/-^ mice.

Although the suppression of monocyte-endothelial cell adhesion and SMC proliferation has been involved in adropin-conferred anti-atherosclerotic effects, the exact mechanism remains unknown [11]. A previous study has documented that adropin can increase the serum level of high density lipoprotein cholesterol and decrease the level of total cholesterol and low density lipoprotein cholesterol in female Wistar rats with hyperlipidemia after a four-week HFD [13]. In this study, however, adropin treatment did not regulate serum lipids in male ApoE^-/-^ mice fed with HFD, which is consistent with the previous study [11]. The disparity may be due to different animal gender and models. In our atherosclerotic models, the adropin treatment did not alter the severity of dyslipidemia. Therefore, the adropin treatment may alleviate atherosclerosis not directly by regulating lipid levels.

EndMT is a fundamental process during development, including the cardiovascular system [3]. Many studies have demonstrated a high incidence of EndMT in a number of inflammation-associated diseases, such as myocardial infarction, and pulmonary hypertension [16–18]. Recent studies have reported that EndMT may induce atherogenic differentiation of endothelial cells [1] and that EndMT may promote atherosclerosis progression [2]. Further evidence suggests that EndMT-derived fibroblast-like cells are common in intimal atherosclerotic plaques [3]. Therefore, the inhibition of EndMT may be an important target to suppress the occurrence and development of atherosclerosis [1–3, 16–18].

Here, we furtherly explored the role of EndMT in adropin-conferred anti-atherosclerosis. Available literature has demonstrated that treating HUVECs with adropin at a dose of 10–25 ng/ml can confer a protective effect [8]. Our use concentrations slightly higher than this that treating HUVECs with adropin at a dose of 30 ng/ml produces a satisfactory inhibitory effect on EndMT. In the current study, the in vivo experiments confirmed the involvement of EndMT by analyzing the altered expression of endothelial and mesenchymal cell markers. With the aortic mRNA directly extracted and collected via repeatedly washing inner cavity of thoracoabdominal aortas with TRIzol Reagent, these markers in aortic tissues were qualitatively analyzed under the confocal microscope and quantitatively assessed by q-PCR. The efficiency of this method was confirmed by the results of q-PCR, that is, adropin treatment reversed HFD-induced decrease in mRNA expression of endothelial cell markers and increase in mRNA expression in mesenchymal cell markers. Furthermore, the adropin-induced alteration in the expression of cell markers was confirmed by analyzing the mRNA and protein expressions of these markers in an in vitro H_2_O_2_-induced EndMT model of HUVECs. The changes in both morphology (smooth muscle-like cells) and phenotype (cell markers) were also directly observed under light and confocal microscopes. In this study, both HUVECs culture in vitro and ApoE^-/-^ mice in vivo confirmed that adropin inhibited the conversion of endothelial cells into SMCs, thus suppressing the EndMT.

In addition, EndMT has been documented to contribute considerably to the vascular remodeling and neointimal formation that arises after a vein graft transplantation into the arterial circulation [19]. This process is dependent on an early activation of the Smad2/3-Snai2 signaling pathway, with antagonism of the TGF-β signaling pathway resulting in decreased EndMT and reduced neointimal formation [19]. It has been demonstrated that TGF-β is the main regulator of the occurrence and development of EndMT [20–22], and that smooth muscle FGF/TGF-β cross talk regulates the atherosclerotic progression [23]. Furthermore, TGF-β receptors bind to the corresponding ligands to form polymer complexes and phosphorylation of Smad in the downstream [19, 24]. The phosphorylated Smad enter the nucleus, activate the nuclear factors, affect the expression of various related genes, and promote the occurrence of EndMT.

In previous studies, the TGF-β/Smad2/3 signaling pathway has not be involved in the processes of adropin-conferred metabolic regulation and endothelial protection, although these processes may involve other signaling pathways, including phosphatidylinositol 3-kinase-Akt [8], extracellular signal regulated kinase 1/2 [8], NB-3/Notch [25], G-protein coupled receptor [26], and proliferator-activated receptor gamma [6, 11]. In this study, we further explored the role of TGF-β/Smad2/3 signaling pathway in H_2_O_2_-treated HUVECs. The involvement of TGF-β was confirmed by the facts that adropin may directly inhibit the gene and/or protein expressions of TGF-β1 and TGF-β2, and that adropin-induced alteration of cell markers is blocked by the transfection of TGF-β1 plasmid. We found that adropin had no significant inhibitory effect on the expression of TGF-βR. Nevertheless, adropin significantly decreased the phosphorylation of Smad2/3, which is the downstream of the TGF-βR pathway. The effects of adropin was blocked by the transfection of TGF-β1 plasmid in this study. These data suggest that the TGF-β/Smad2/3 pathway may be involved in adropin-induced attenuation of EndMT.

Some limitations remain in this study. First, only single dose of adropin was in vivo and in vitro used in this study. Second, the inhibitors of TGF-β/Smad were not used in this study, although the use of TGF-β1 plasmid helped explore the mechanism. Third, we did not involve noncanonical signaling pathways which also have a bearing on TGF β- Smad cascade. Fourth, we did not explore other mechanisms underlying the effect of adropin on AS. Fifth, in vitro cell models cannot fully simulate in vivo animal models.

In conclusion, adropin can reduce atherosclerosis in ApoE^-/-^/Enho^-/-^ mice through non-lipid-regulating pathway, by inhibiting EndMT via the TGF-β/Smad2/3 signaling pathway. The findings signify the potential therapeutic value of adropin in the prevention of atherosclerosis.

## Materials and methods

This study was reviewed and approved by the Experimental Animal Care Committee of Fujian Medical University Union Hospital and the experimental procedures observed the Guide for the Care and Use of Laboratory Animals revised by the National Institutes of Health in 2011.

### In vivo animal atherosclerotic models and experimental protocols

Specific pathogen free grade male ApoE^-/-^ and DKO mice (aged 8 weeks old and weighed 18g–25g) were respectively purchased from Beijing Huafukang Biotechnology Co., Ltd (China) and Beijing Viewsolid Biotechnology Co., Ltd (China). The animals were housed in a conventional animal facility. To establish the models of atherosclerosis, ApoE^-/-^ and DKO mice were fed with HFD (2% cholesterol, 9% lard, 0.2% bile extract, 5% yolk powder, 5% whole milk powder and 78.8% basic feed) for 13 weeks as described previously [12]. All mice were divided into five groups (*n* = 10 mice/group) (Supplementary Fig. 1): (1) Normal diet (ND) group, in which the ApoE^-/-^ mice were fed with normal chow diet; (2) HFD group, with ApoE^-/-^ mice fed with HFD; (3) HFD+Adropin group, in which the ApoE^-/-^ mice received HFD and were intraperitoneally injected with adropin (105 μg/kg) (Phoenix Pharmaceuticals, USA) every day for 13 weeks; (4) DKO-HFD group, with the DKO mice fed with HFD; (5) DKO-HFD+Adropin group, in which the DKO mice received HFD and an intraperitoneal injection of adropin.

At the end of the experiments, all animals were anesthetized with an intraperitoneal injection of ketamine (75 mg/kg) and diazepam (7.5 mg/kg). Afterwards, the tissue and blood samples were collected as follows (Supplementary Fig. 1): (1) venous blood samples were collected from five ApoE^-/-^ mice per group for the assessment of biochemical indexes. Their aortic roots were dissected to evaluate the severity of atherosclerosis by Oil Red O (ORO) and haematoxylin-eosin (H&E) staining. The expression of EndMT-related markers was detected by immunofluorescence. The mRNA of aortic inner wall cells (mainly endothelial cells) was collected by repeatedly washing the inner cavity of thoracoabdominal aortas with TRIzol Reagent (Invitrogen, CA, USA). The mRNA expression of EndMT-related markers was measured by quantitative real-time PCR (q-PCR); (2) the aortic roots from five DKO mice per group were dissected for ORO and H&E staining; (3) The whole aortas (including the ascending arch, thoracic and abdominal segments) were dissected from another five animals per group and stained with ORO to further assess the severity of atherosclerotic lesions.

### In vitro endothelial-to-mesenchymal transition models and experimental protocols

HUVECs were purchased from ScienCell Research Laboratories (Carlsbad, CA, USA) and were cultured in a 5% CO_2_ atmosphere at 37 °C with an endothelial cell medium (Cat No.1001, ScienCell, USA) containing 5% fetal bovine serum (Cat No.0025, ScienCell, USA), 1% endothelial cell growth supplement (Cat No.1052, ScienCell, USA) and 100 U/mL penicillin-streptomycin (Cat No.0503, ScienCell, USA).

To establish the in vitro EndMT model, HUVECs were treated with 100 μmol/L H_2_O_2_ for 6 days as described previously [3], and divided into four groups: (1) Control group, with no further treatments; (2) H_2_O_2_ group, with cells treated with H_2_O_2_; (3) H_2_O_2_ + Adropin group, in which cells were incubated with 30 ng/ml adropin 10 min before the H_2_O_2_ treatment; (4) H_2_O_2_ + Adropin+TGF-β plasmid group, receiving the same treatment as the H_2_O_2_ + Adropin group, as well as the cellular transfection of transforming growth factor (TGF)-β1 plasmid.

The TGF-β1 plasmid was amplified in Escherichia coli (Addgene, USA) and then extracted by plasmid isolation with the EasyPure^®^ HiPure Plasmid MaxiPrep Kit (TransGen Biotech, China) according to the manufacturer’s protocol. For the transfection (at a density of 2 × 10^5^ HUVECs per well), the DNA of TGF-β1 plasmid (0.8 μg) was diluted in 50 μl of a serum-free medium and placed in a resting state for 5 min. Lipofectamine 2000 (2 μl) was diluted in 48 μl of the serum-free medium and left in a resting state for 5 min. Afterwards, the diluted DNA was combined with the diluted Lipofectamine 2000 (total volume = 100 μl) and incubated at room temperature for 20 min. Next, the 100 μl of complexes were added to each well. Subsequently, the cells were further incubated at 37 °C in a CO_2_ chamber for 24–48 h prior to transgenic expression testing.

After a 6-day culture, HUVECs were collected to assess the effects of adropin on H_2_O_2_-induced EndMT under light and confocal microscopes. The expressions of CD31, VE-cadherin, α-SMA, TGF-β1, and TGF-β2 in HUVECs were assessed by q-PCR and Western blotting. The expressions of TGF-βR and Smad2/3 were respectively detected by q-PCR and by Western blotting.

### Oil Red O staining and Hematoxylin-Eosin staining

To visualize atherosclerotic lesions and determine plaque size, consecutive frozen sections from aortic roots were treated by ORO and H&E staining (Sigma-Aldrich, USA), and the whole aortas by ORO staining, as described previously [2, 27]. In brief, for ORO staining, the whole aortas were incubated with a fresh ORO solution for 30 min; frozen sections were incubated with a fresh ORO solution for 30 min and hematoxylin for 3 min. Lipid droplets in atherosclerotic lesions were stained red, and nuclei blue. For H&E staining, frozen sections were incubated with a fresh hematoxylin solution for 12 min and the eosin solution for 30 s, and the nuclei were stained hyacinthine, and cytoplasm red. The images were analyzed and quantified with Image Pro Plus 6.0 software. In the whole aortas, the surface area of the atherosclerotic lesion was quantified and expressed as mm^2^, as described previously [2]. In the frozen sections, the results were expressed as a percentage of lipid area to atherosclerotic plaque area by ORO staining, and a percentage of atherosclerotic plaque area to vascular lumen area by H&E staining.

### Immunofluorescence

To evaluate the extent of EndMT, the expressions of CD31 and SM22α in aortic roots from the ApoE^-/-^ mice and α-SMA in the H_2_O_2_-treated HUVECs were determined by immunofluorescence. Consecutive frozen aortic sections used in ORO and H&E staining were also used in immunofluorescence. The frozen aortic sections and HUVECs were fixed with 4% paraformaldehyde, permeabilized with 0.5% Triton X-100, and blocked in 2% bovine serum albumin for 30 min at room temperature. CD31, SM22α and α-SMA were treated their primary antibodies and their expressions were examined using anti-Rabbit or anti-Mouse IgG. After a 2-min staining with 4’,6-diamidino-2-phenylindole (DAPI), the fluorescent images were taken under a confocal laser scanning microscope (Thermo, USA). Qualitative analysis was performed for aortic tissues and quantitative analysis for cultured HUVECs. The α-SMA positive HUVECs were calculated and expressed as a percentage over the total number of DAPI positive nuclei in the field of the confocal microscope. Smooth muscle-like cells, which occurred due to H_2_O_2_-induced morphological changes, were measured and expressed as a percentage relative to the total number of HUVECs in the field of the light microscope. All antibodies used in this study are summarized and listed in Supplementary Table 1.

### Quantitative real-time PCR

q-PCR was performed to detect the mNRA expressions of CD31, VE-cadherin, α-SMA, and fibroblasts specific protein-1 (FSP-1) in the aortic inner wall, and those of CD31, VE-cadherin, α-SMA, TGF-β1, TGF-β2, and TGF-βR in the HUVECs.

Trizol reagent (Invitrogen, CA, USA) was applied to extract the total RNA from the aortic inner wall (intima) and HUVECs. The extracted RNA samples were converted into first strand cDNA using an RT2 First Strand kit, following the manufacturer’s protocol (Qiagen, Germany). Quantitative gene expression was analyzed by q-PCR and performed on an ABI Prism 7900 HT (Applied Biosystems, Foster City, CA). Gene specific primers (Qiagen, Germany) and SYBR Green q-PCR detection kit (Qiagen, Germany) were employed as instructed by the manufacturer. Data were analyzed by the 2^-ΔΔCt^ method. Primers used are summarized in Supplementary Table 2.

### Western blotting

Western blotting was performed to determine the protein expressions of CD31, VE-cadherin, α-SMA, TGF-β1, TGF-β2, and the total and phosphorylated protein of Smad2/3 in HUVECs. Cells were lysed with a buffer (containing 20 mmol/L Tris, 150 mmol/L NaCl, 1 mmol/L EDTA, 1 mmol/L EGTA, 1% Triton × 100, 2.5 mmol/L sodium pyrophosphate, 1 mmol/L β-glycerophosphate, 1 mmol/L Na_3_VO_4_, 50 mmol/L NaF, protease inhibitor cocktail; pH 7.5; Beyotime; phosphatase inhibitor cocktail, Roche). The concentrations of the samples were determined with a BCA kit (Beyotime, China), with the bovine serum albumin (BSA) as a reference protein. The samples were separated by SDS-PAGE and transferred to nitrocellulose membranes, followed by incubation with primary antibodies at 4 °C overnight. After an incubation with the fluorescence-conjugated secondary antibodies (Santa Cruz Biotechnology, China) at room temperature for 1 h, the immunoreactive proteins were visualized with a Chemiluminescence gel imager (Tanon-5500) (Biotanon, shanghai, CA). Data were normalized by setting the densitometry of control sample at 1. All antibodies used in this study are listed in Supplementary Table 1.

### Statistical analyses

The measurement data were expressed by mean ± SEM. Statistical analysis was performed with SPSS 20.0 for Windows. For comparison between multiple groups, data were analyzed by *t* tests or ANOVA. When a statistical difference appeared, the least significant difference procedure was applied. A value of *P* < 0.05 was considered statistically significant.

### Supplementary information

Supplementary Table 1

Supplementary Table 2

Supplementary Figure Legends

Supplementary Fig. 1

Supplementary Fig. 2

Original Data File

## Data Availability

The corresponding author will provide the original data used to support the findings of this study upon reasonable request. Please direct inquiries to the corresponding author.

## References

[1] Moonen JR, Lee ES, Schmidt M, Maleszewska M, Koerts JA, Brouwer LA (2015). Endothelial-to-mesenchymal transition contributes to fibro-proliferative vascular disease and is modulated by fluid shear stress. Cardiovasc Res.

[2] Chen PY, Qin L, Baeyens N, Li G, Afolabi T, Budatha M (2015). Endothelial-to-mesenchymal transition drives atherosclerosis progression. J Clin Investig.

[3] Evrard SM, Lecce L, Michelis KC, Nomura-Kitabayashi A, Pandey G, Purushothaman KR (2016). Endothelial to mesenchymal transition is common in atherosclerotic lesions and is associated with plaque instability. Nat Commun.

[4] Krenning G, Moonen JR, van Luyn MJ, Harmsen MC (2008). Vascular smooth muscle cells for use in vascular tissue engineering obtained by endothelial-to-mesenchymal transdifferentiation (EnMT) on collagen matrices. Biomaterials.

[5] Moonen JR, Krenning G, Brinker MG, Koerts JA, van Luyn MJ, Harmsen MC (2010). Endothelial progenitor cells give rise to pro-angiogenic smooth muscle-like progeny. Cardiovasc Res.

[6] Kumar KG, Trevaskis JL, Lam DD, Sutton GM, Koza RA, Chouljenko VN (2008). Identification of adropin as a secreted factor linking dietary macronutrient intake with energy homeostasis and lipid metabolism. Cell Metab.

[7] Kumar KG, Zhang J, Gao S, Rossi J, McGuinness OP, Halem HH (2012). Adropin deficiency is associated with increased adiposity and insulin resistance. Obesity.

[8] Lovren F, Pan Y, Quan A, Singh KK, Shukla PC, Gupta M (2010). Adropin is a novel regulator of endothelial function. Circulation.

[9] Li H, Hu D, Chen G, Zheng D, Li S, Lin Y (2021). Adropin-based dual treatment enhances the therapeutic potential of mesenchymal stem cells in rat myocardial infarction. Cell Death Dis.

[10] Wu L, Fang J, Chen L, Zhao Z, Luo Y, Lin C (2014). Low serum adropin is associated with coronary atherosclerosis in type 2 diabetic and non-diabetic patients. Clin Chem Lab Med.

[11] Sato K, Yamashita T, Shirai R, Shibata K, Okano T, Yamaguchi M (2018). Adropin contributes to anti-atherosclerosis by suppressing monocyte-endothelial cell adhesion and smooth muscle cell proliferation. Int J Mol Sci.

[12] Okutsu M, Lira VA, Higashida K, Peake J, Higuchi M, Suzuki K (2014). Corticosterone accelerates atherosclerosis in the apolipoprotein E-deficient mouse. Atherosclerosis.

[13] Akcılar R, Emel Koçak F, Şimşek H, Akcılar A, Bayat Z, Ece E (2016). The effect of adropin on lipid and glucose metabolism in rats with hyperlipidemia. Iran J Basic Med Sci.

[14] Gao S, McMillan RP, Jacas J, Zhu Q, Li X, Kumar GK (2014). Regulation of substrate oxidation preferences in muscle by the peptide hormone adropin. Diabetes.

[15] Gao S, McMillan RP, Zhu Q, Lopaschuk GD, Hulver MW, Butler AA (2015). Therapeutic effects of adropin on glucose tolerance and substrate utilization in diet-induced obese mice with insulin resistance. Mol Metab.

[16] Chen PY, Schwartz MA, Simons M (2020). Endothelial-to-mesenchymal transition, vascular inflammation, and atherosclerosis. Front Cardiovasc Med.

[17] Kovacic JC, Dimmeler S, Harvey RP, Finkel T, Aikawa E, Krenning G (2019). Endothelial to mesenchymal transition in cardiovascular disease: JACC state-of-the-art review. J Am Coll Cardiol.

[18] Mehta V, Pang KL, Givens CS, Chen Z, Huang J, Sweet DT (2021). Mechanical forces regulate endothelial-to-mesenchymal transition and atherosclerosis via an Alk5-Shc mechanotransduction pathway. Sci Adv.

[19] Cooley BC, Nevado J, Mellad J, Yang D, St Hilaire C, Negro A (2014). TGF-beta signaling mediates endothelial-to-mesenchymal transition (EndMT) during vein graft remodeling. Sci Transl Med.

[20] Guihard PJ, Yao J, Blazquez-Medela AM, Iruela-Arispe L, Boström KI, Yao Y (2016). Endothelial-mesenchymal transition in vascular calcification of Ins2Akita/+ Mice. PLoS One.

[21] ten Dijke P, Arthur HM (2007). Extracellular control of TGF-beta signalling in vascular development and disease. Nat Rev Mol Cell Biol.

[22] Guo Y, Li P, Bledsoe G, Yang ZR, Chao L, Chao J (2015). Kallistatin inhibits TGF-β-induced endothelial-mesenchymal transition by differential regulation of microRNA-21 and eNOS expression. Exp Cell Res.

[23] Chen PY, Qin L, Li G, Tellides G, Simons M (2016). Smooth muscle FGF/TGFbeta cross talk regulates atherosclerosis progression. EMBO Mol Med.

[24] Wu M, Peng Z, Zu C, Ma J, Lu S, Zhong J (2016). Losartan attenuates myocardial endothelial-to-mesenchymal transition in spontaneous hypertensive rats via inhibiting TGF-β/Smad signaling. PLoS One.

[25] Wong CM, Wang Y, Lee JT, Huang Z, Wu D, Xu A (2014). Adropin is a brain membrane-bound protein regulating physical activity via the NB-3/NOTCH signaling pathway in mice. J Biol Chem.

[26] Thapa D, Stoner MW, Zhang M, Xie B, Manning JR, Guimaraes D (2018). Adropin regulates pyruvate dehydrogenase in cardiac cells via a novel GPCR-MAPK-PDK4 signaling pathway. Redox Biol.

[27] Andrés-Manzano MJ, Andrés V, Dorado B (2015). Oil Red O and hematoxylin and eosin staining for quantification of atherosclerosis burden in mouse aorta and aortic root. Methods Mol Biol.

