# Supplementary material for: Adropin inhibits the progression of atherosclerosis in ApoE-/-/Enho-/- mice by regulating endothelial-to-mesenchymal transition
Source: Cell Death Discov. 2023 Oct 31;9:402. doi: 10.1038/s41420-023-01697-3 (PMC10616072; doi:10.1038/s41420-023-01697-3)
Supplement: Supplementary file 1 — Supplementary Table 1 [file 41420_2023_1697_MOESM1_ESM.docx]

**Table. 1 Antibodies used in this study**

| **Antigen** | **Source (product number)** | **Host** | **Dilution** |
| --- | --- | --- | --- |
| Anti SM22α | Proteintech(60213-1-lg) | mouse | 1:1000 |
| Anti α-SMA | Abcam(ab5694) | rabbit | 1:1000 |
| CD31 | Abcam(ab28364) | rabbit | 1:1000 |
| VE-cadherin | Abcam(ab33168) | rabbit | 1:1000 |
| TGF-B1 | Abcam(ab64715) | mouse | 1:1000 |
| TGF-B2 | Abcam(ab36495) | mouse | 1:1000 |
| P-SMAD2/3 | cell signaling(8828) | rabbit | 1:1000 |
| SMAD2/3 | cell signaling(8685) | rabbit | 1:1000 |
| GAPDH | Proteintech(HRP-60004) |  | 1:5000 |
