# Supplementary material for: Adropin inhibits the progression of atherosclerosis in ApoE-/-/Enho-/- mice by regulating endothelial-to-mesenchymal transition
Source: Cell Death Discov. 2023 Oct 31;9:402. doi: 10.1038/s41420-023-01697-3 (PMC10616072; doi:10.1038/s41420-023-01697-3)
Supplement: Supplementary file 2 — Supplementary Table 2 [file 41420_2023_1697_MOESM2_ESM.docx]

**Table. 2 Sequences of primers used in quantitative q-PCR assay.**

| mRNA | Species | Forward primer Sequence (5'->3') | Reverse Primer Sequence (5'->3') |
| --- | --- | --- | --- |
| CD31 | Mice | ACGCTGGTGCTCTATGCAAG | TCAGTTGCTGCCCATTCATCA |
| VE-cadherin | Mice | TCAACGCATCTGTGCCAGAGAT | CACGATTTGGTACAAGACAGTG |
| α-SMA | Mice | TGACAATGGCTCTGGGCTCTGTAA | TTCGTCACCCACGTAGCTGTCTTT |
| FSP-1 | Mice | TCCACAAATACTCAGGCAAAGAG | GCAGCTCCCTGGTCAGTAG |
| GAPDH | Mice | AGGTCGGTGTGAACGGATTTG | TGTAGACCATGTAGTTGAGGTCA |
| CD31 | Humen | GAGTCCAGCCGCATATCC | TGACACAATCGTATCTTCCTTC |
| VE-cadherin | Humen | CAGCCCAAAGTGTGTGAGAA | TGTGATGTTGGCCGTGTTAT |
| α-SMA | Humen | TGACAATGGCTCTGGGCTCTGTAA | TTCGTCACCCACGTAGCTGTCTTT |
| FSP-1 | Humen | GTCCACCTTCCACAAGTAC | TGTCCAAGTTGCTCATCAG |
| TGF-β1 | Humen | TGACAAGGCCCATGATGGTT | GACTCCTGCTGATTCCCCAC |
| TGF-β2 | Humen | GCGACGAAGAGTACTACGCC | ATGGCATCAAGGTACCCACAG |
| TGF-βR | Humen | TCCAACTACTGGTTTACCATTGC | ACAGCAACTTCTTCTCCCCG |
| GAPDH | Humen | GGAGCGAGATCCCTCCAAAAT | GGCTGTTGTCATACTTCTCATGG |
