# Supplementary material for: Adropin inhibits the progression of atherosclerosis in ApoE-/-/Enho-/- mice by regulating endothelial-to-mesenchymal transition
Source: Cell Death Discov. 2023 Oct 31;9:402. doi: 10.1038/s41420-023-01697-3 (PMC10616072; doi:10.1038/s41420-023-01697-3)
Supplement: Supplementary file 3 — Supplementary Figure Legends [file 41420_2023_1697_MOESM3_ESM.docx]

**Supplemental Figure Legends**

**Supplemental Figure 1**

*In vivo* animal atherosclerotic models and experimental protocols. ApoE^-/-^, apolipoprotein E knockout mice; ND, normal diet; HFD, high fat diet; ORO, oil Red O; H&E, haematoxylin-eosin; mRNA, messenger ribonucleic acid; ApoE^-/-^/Enho^-/-^, apolipoprotein E and energy homeostasis-associated gene double knockout; DKO, double gene knockout.

**Supplemental Figure 2**

Blood lipid profile and body weight in ApoE^-/-^ mice by Adropin treatment. A and B: Blood lipid profile in ApoE^-/-^ mice. C and D: body weight in ApoE^-/-^ mice. The data are presented as mean ± SEM. **P*<0.05, ***P*<0.01, ****P*<0.001, & *P*<0.05, &&*P*<0.01, &&&*P*<0.001.

**Supplemental Figure 3**

The full and uncropped western blot of CD31, VE-cadherin, α-SMA, TGF-β1, TGF-β2, p-Smad2/3 and p-Smad2/3.
