# Supplementary figures and images for: Adropin inhibits the progression of atherosclerosis in ApoE-/-/Enho-/- mice by regulating endothelial-to-mesenchymal transition
Source: Cell Death Discov. 2023 Oct 31;9:402. doi: 10.1038/s41420-023-01697-3 (PMC10616072; doi:10.1038/s41420-023-01697-3)

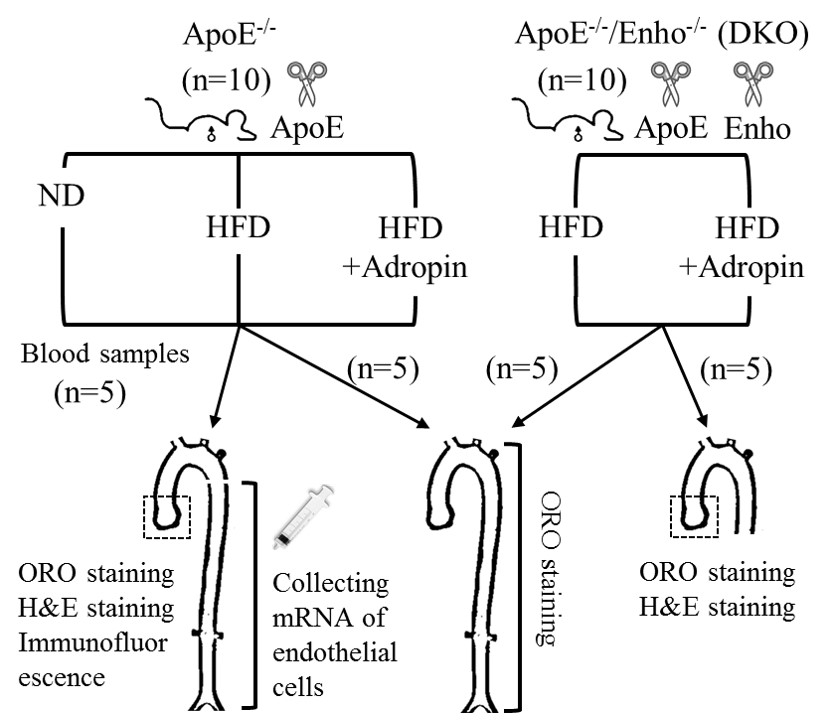

Supplement: Supplementary file 4 — Supplementary Fig. 1 [file 41420_2023_1697_MOESM4_ESM.jpg]

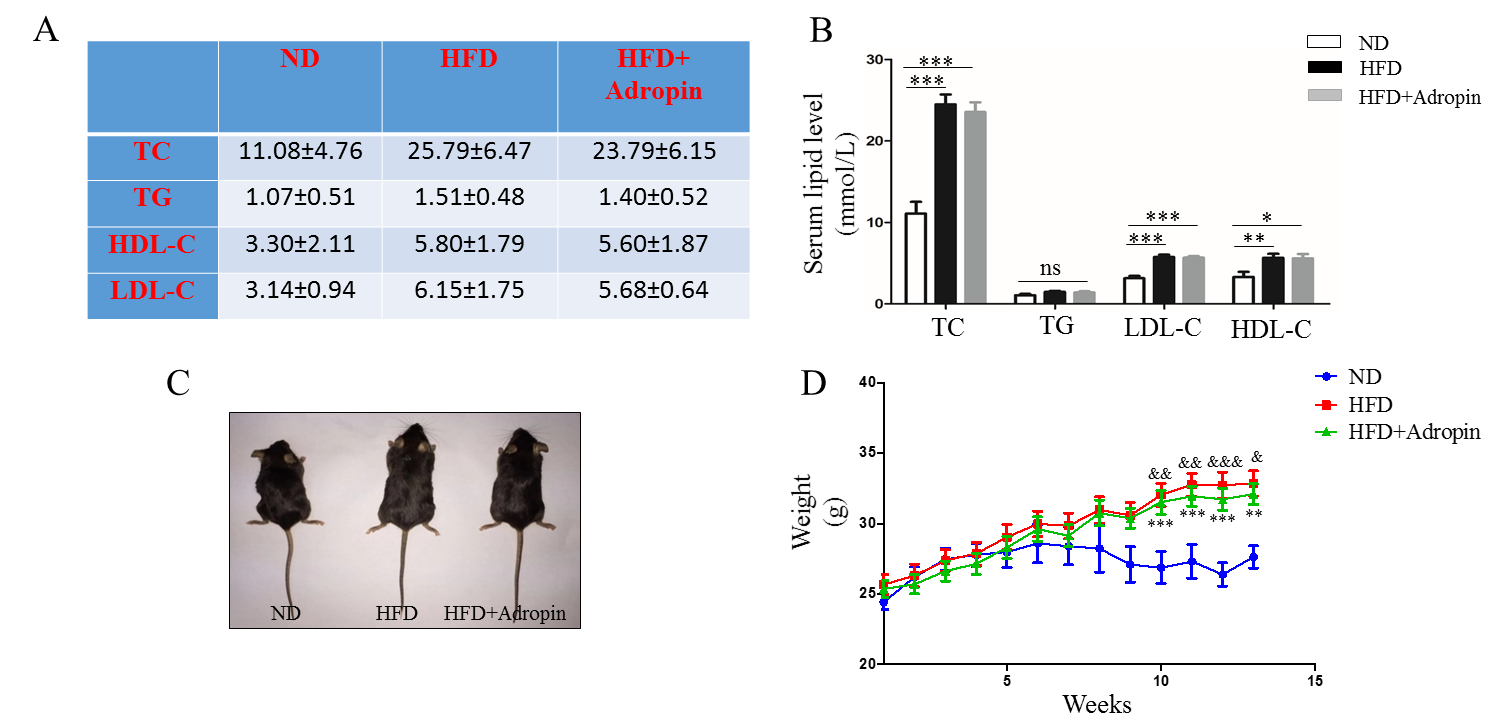

Supplement: Supplementary file 5 — Supplementary Fig. 2 [file 41420_2023_1697_MOESM5_ESM.tif]

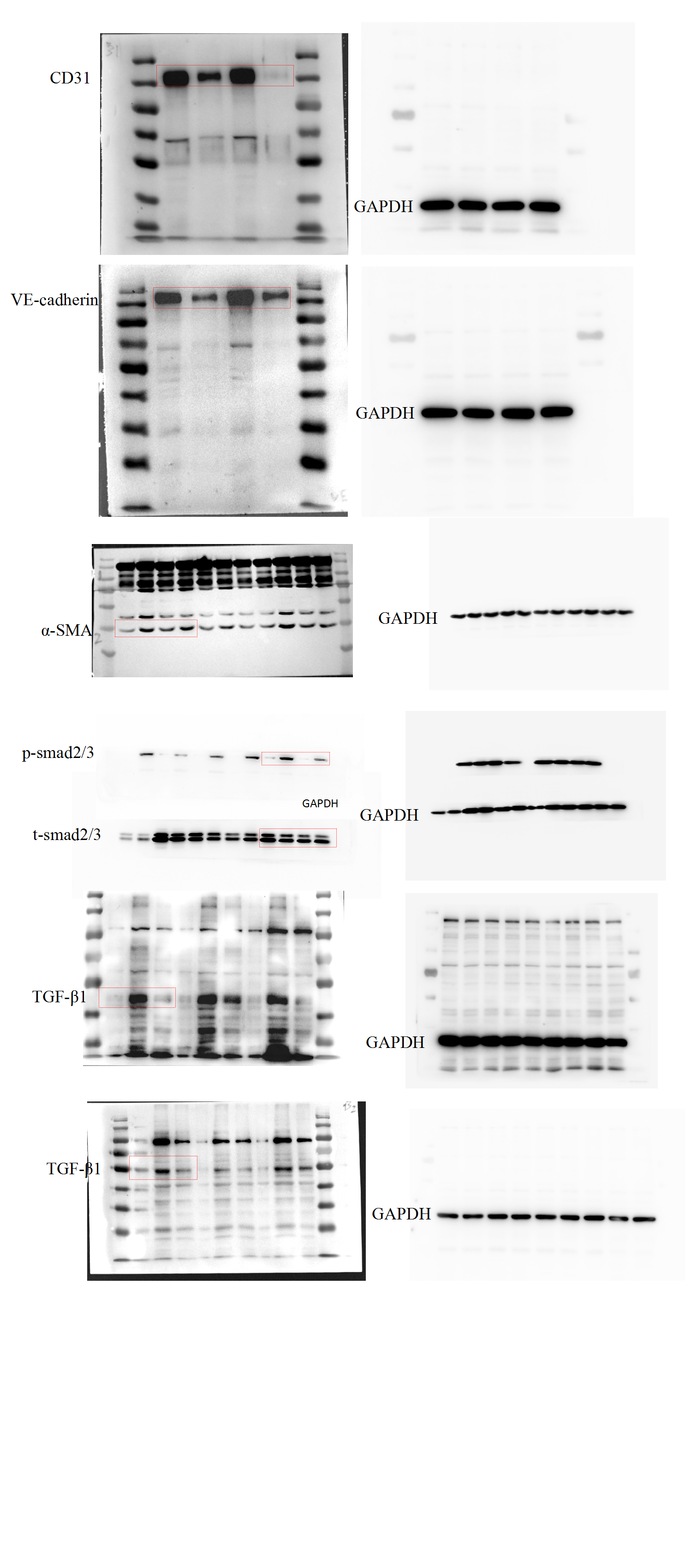

Supplement: Supplementary file 6 — Original Data File [file 41420_2023_1697_MOESM6_ESM.tif]
